# 5.2 RETHINKING THE COURSE OF PSYCHOTIC DISORDERS: IDENTIFYING LATENT TRAJECTORIES

**DOI:** 10.1093/schbul/sby014.014

**Published:** 2018-04-01

**Authors:** Craig Morgan, Paola Dazzan, Julia Lappin, Margaret Heslin, Gillian Doody, Robin Murray, Peter B Jones, Ulrich Reininghaus

**Affiliations:** 1 Institute of Psychiatry, Psychology & Neuroscience, King’s College London; 2 University of New South Wales; 3 University of Nottingham; 4 University of Cambridge; 5 Maastricht University

## Abstract

**Background:**

The clinical course of psychotic disorders is highly variable. Typically, researchers have captured course types using broad categories, e.g. the WHO instruments to assess course and outcome distinguish three categories: episodic (i.e., no episode > 6 months), continuous (i.e., no remission > 6 months), and neither (i.e., an episode and a remission > 6 months). However, whether these adequately capture symptom trajectories of psychotic disorders has not been assessed. Using AESOP-10 data, we sought to identify classes of individuals with specific symptom trajectories over a 10 year follow up and to, then, compare trajectories with WHO categories and examine associations between trajectories and baseline demographic characteristics and diagnoses.

**Methods:**

AESOP-10 is a follow-up, at 10 years, of a cohort of 552 patients with a first episode psychosis identified in south-east London and Nottingham, UK. At follow-up, we collated detailed information on clinical and social course and outcome. This included collating extensive information on month by month fluctuations in presence of psychotic symptoms. Using this data, we fitted growth mixture models to identify latent trajectory classes that accounted for heterogeneity in patterns of change in psychotic symptoms over time.

**Results:**

We had sufficient data on occurrence of psychotic symptoms throughout the follow up on 326 (~ 60%) patients.

A four-class quadratic growth mixture model best fit the data, with four trajectories defined by variations in the mean number of months psychotic per year during the follow-up period: (1) low and reducing [intermittent] (58.5%); (2) persistently high [persistent] (30.6%); (3) high, followed by gradual reduction [late improvement] (5.6%); and (4) intermediate, followed by gradual increase [late decline] (5.4%). When compared with the usual classification of course types (episodic, continuous, neither), the intermittent class included all those in the episodic category (n 94 of 94; 100%) and the persistent class included almost all those in the continuous category (n 72 of 78 persistent; 92%). A majority of those in the neither category had an intermittent trajectory (i.e., n 90 of 145; 62%), with the remainder spread across the other three classes (i.e., n 25, 17% persistent; n 14, 10% late decline; n 16, 11% late improvement).

Compared with those with an intermittent trajectory, patients with a persistent trajectory were less often women (OR 0.6, 95% CI 0.4–0.9), more often of black Caribbean ethnicity (OR 2.3, 95% CI 1.2–4.1), and less often had a diagnosis of affective psychosis (OR 0.2, 95% CI 0.1–0.4). There were no differences by age. Numbers were small, but there were indications that those with a late decline trajectory more closely resembled those with a persistent trajectory (i.e., less often women, more often of black Caribbean ethnicity, less often diagnosis of affective psychosis) than did those with a late improvement trajectory.

**Discussion:**

Our current approach to classifying course of psychotic disorders may be flawed, particularly in specifying a group as neither episodic nor continuous. Our findings suggest this group is heterogeneous and includes patients whose outcomes more closely resemble one of the two main trajectories, intermittent or persistent. Only a small proportion of patients fit neither. These patients constitute clinically important sub-groups whose trajectories appear to change, either from an initially positive or initially negative course, some years after first contact with mental health services. Our failure to fully characterise trajectories of psychosis may confound efforts to elucidate predictors of long-term outcome.

